# DroughtDB: an expert-curated compilation of plant drought stress genes and their homologs in nine species

**DOI:** 10.1093/database/bav046

**Published:** 2015-05-15

**Authors:** Svenja Alter, Kai C. Bader, Manuel Spannagl, Yu Wang, Eva Bauer, Chris-Carolin Schön, Klaus F. X. Mayer

**Affiliations:** ^1^Plant Breeding, Center of Life and Food Sciences Weihenstephan, Technische Universität München, 85354 Freising, Germany, ^2^Department of Plant Genome and Systems Biology, Helmholtz Center Munich, German Research Center for Environmental Health, 85764 Neuherberg, Germany and ^3^College of Science, King Saud University, Riyadh 11451, Kingdom of Saudi Arabia

## Abstract

Plants are sessile and therefore exposed to a number of biotic and abiotic stresses. Drought is the major abiotic stress restricting plant growth worldwide. A number of genes involved in drought stress response have already been characterized, mainly in the model species *Arabidopsis thaliana* and *Oryza sativa.* However, with the aim to produce drought tolerant crop varieties, it is of importance to identify the respective orthologs for each species. We have developed DroughtDB, a manually curated compilation of molecularly characterized genes that are involved in drought stress response. DroughtDB includes information about the originally identified gene, its physiological and/or molecular function and mutant phenotypes and provides detailed information about computed orthologous genes in nine model and crop plant species including maize and barley. All identified orthologs are interlinked with the respective reference entry in MIPS/PGSB PlantsDB, which allows retrieval of additional information like genome context and sequence information. Thus, DroughtDB is a valuable resource and information tool for researchers working on drought stress and will facilitate the identification, analysis and characterization of genes involved in drought stress tolerance in agriculturally important crop plants.

**Database URL:** http://pgsb.helmholtz-muenchen.de/droughtdb/

## Introduction

Drought is one of the major constraints of plant productivity worldwide and for the next 30–90 years severe, widely spread drought scenarios are predicted for large parts of land areas (1). The predicted climate change and the increasing world population lead to a growing demand for water and reveal the urgent need for drought tolerant crops. A detailed understanding of the molecular and physiological processes and regulatory networks in crop plants exposed to drought stress will be instrumental for genome-based breeding and development of drought tolerant crop varieties.

When exposed to reduced water availability plants exhibit various physiological responses. A pivotal reaction is stomatal closure to avoid water loss by transpiration. The resulting reduced availability of carbon dioxide together with a down regulation of photosynthesis-related genes lead to a decrease in carbon assimilation restricting plant growth and productivity. Furthermore, an increase in photorespiration under drought stress conditions leads to an accumulation of reactive oxygen species (ROS), which are toxic for cellular components and will eventually lead to cell death (2). To cope with reduced water availability plants have evolved a number of physiological and molecular adaptation mechanisms, which can be categorized into drought avoidance and drought tolerance (3). A dehydration avoidance mechanism contributing to reduced transpiration rates is for example leaf rolling, which generates a favorable microclimate and reduces the effective leaf area exposed to radiation (4, 5). Another example for drought avoidance is the increase of the root to shoot ratio, which is caused by roots being less sensitive to drought-induced growth inhibition than shoots (6). On the other hand, plants accumulate osmolytes that act as compatible solutes and lead to a decrease of the osmotic potential. The resulting drought tolerance can be mediated by amino acids like proline, organic acids, sugar alcohols and sugars by acting as compatible solutes to maintain cellular functions (7).

Transcriptomic approaches have revealed thousands of genes that are differentially regulated upon drought stress. According to Deyholos (8) more than 100 studies from 28 plant species had been published by the year 2010 which focused toward drought or salinity stress response. These experiments mainly used microarray platforms whereas within the last 5 years mainly next generation sequencing approaches (e.g. RNA-Seq) were carried out and broadened our knowledge about gene expression networks under stress conditions. Databases dedicated to stress-responsive genes exist which comprise genes that are differentially regulated under stress conditions. The Arabidopsis Stress Responsive Gene Database contains 636 genes from *Arabidopsis thaliana* (*Arabidopsis*) in the context of 44 different stress conditions including abiotic and biotic stresses (9). The Stress-Responsive Transcription Factor DataBase (STIFDB) is as well a collection of abiotic and biotic stress responsive genes from *Arabidopsis* and *Oryza sativa* (rice) including the possibility to identify putative binding sites in the promoters of these genes (10, 11). In the same line, there is a database of rice transcription factors under stress conditions, RiceSRTFDB, giving information about expression, *cis*-elements and information about mutant phenotypes (12). Moreover, there is a general ‘Plant Stress Gene Database’ (13) including 259 stress-related genes from 11 species and for some genes also information about orthologs is included. However, all these databases are neither specific for drought stress nor dedicated to genes with experimentally proven function under stress conditions. Usually, comprehensive expression data are used for the final identification of genes that contribute to drought tolerance (8). The products of these genes can directly contribute to drought tolerance for example as protection factors or as enzymes for osmolyte biosynthesis. Furthermore, many of these genes encode transcription factors that control genes, which are important for diverse physiological responses to drought stress. To ultimately demonstrate a functional role of a gene in drought tolerance a functional characterization by analysis of loss-of-function mutants or overexpressing lines has to be performed. In the recent past, an increasing number of these genes have been characterized and their function under drought conditions has been demonstrated. Most of these functional studies have been undertaken in the eudicot model species *A.* *thaliana* and in the grass *O.* *sativa.* However, a prerequisite for the production of drought-tolerant crop species is the identification of the respective orthologous genes in each species. Owing to the rapid progress in transcriptome and whole-genome sequencing of different plant species due to the advancements in sequencing technologies it is has now become feasible to reliably identify orthologous genes across several model and crop species.

Our aim was to generate a manually curated database with drought stress genes that have an experimentally verified function in drought tolerance and to provide computed orthologous genes available from whole-genome sequencing projects. We performed an extensive literature research and identified 199 molecularly characterized genes from various plant species under drought stress from a total of 182 publications. These genes served as entry for the drought gene database. The availability of genomic sequences from recently completed whole-genome sequencing projects, namely from *Sorghum bicolor* (sorghum) (14), *Zea mays* (maize) (15), *Brachypodium distachyon* (Brachypodium) (16), *Solanum lycopersicum* (tomato) (17), *Hordeum vulgare* (barley) (18), *Aegilops tauschii* (19) and from an RNA-Seq resource from *Secale cereale* (rye) (20) allowed us to identify orthologous genes in these crops as well as in *Arabidopsis* and rice. This database can serve as an information resource and tool for researchers to gather information about orthologs of drought stress associated genes in nine plant species. It will help to expand the analysis of molecular drought response toward crops and will allow to analyse and interpret new genome scale experimental findings in a comparative context.

### Data generation and database content

We manually screened the literature for genes that have been molecularly characterized and retrieved information about their respective function *in planta.* A total of 199 molecularly characterized genes from 38 plant species under drought stress conditions were included as basic entities of the database. We define ‘molecularly characterized’ as follows: a gene has been identified (or cloned) as related to drought stress response in one plant species by characterizing (i) mutants of the gene that show an altered response to drought stress as compared with the wild type or (ii) transgenic plants, typically *Arabidopsis*, rice or *Nicotiana tabacum* (tobacco), showing an altered tolerance against drought stress when overexpressing this gene. As an example, *GhMT3a,* a gene from cotton encoding a metallothionein which acts as a ROS scavenger, has been overexpressed in transgenic tobacco plants, resulting in an increased tolerance against drought stress compared with wild-type plants (21).

For all 199 molecularly characterized genes, orthologous groups were constructed using the gene contents of *Arabidopsis*, rice, sorghum, maize, Brachypodium, tomato, barley, rye and *Aegilops tauschii* (as a representation for wheat). Hereby, OrthoMCL version 2.0 (http://orthomcl.org/) (22) was used to not only to identify gene families shared by multiple species but also species- or lineage-specific gene family expansions. OrthoMCL gene family clusters are characterized as orthologous groups containing proteins related by either orthology (representing recent descent), inparalology (representing recent duplication) or co-orthology (representing recent descent and duplication). Essentially, all these relationships among proteins from the nine plant species outlined above were calculated based on sequence homology.

All genes involved in drought stress response were classified with respect to genes involved in physiological adaptation and/or molecular adaptation after application of drought stress to the plant. Further down, genes are grouped into more detailed pathways. Protein sequences were extracted from various public databases for the drought stress-related genes and deposited in the database.

### Database design

The database was set up in different access and navigation layers to facilitate intuitive user access.

Layer 1 consists of two different blocks where the first block describes physiological adaptation. Upon sensing water shortage, plants in general show three major adaptive responses (23): (i) reestablishment of ion and osmotic homeostasis, (ii) growth control and (iii) detoxification signaling. For all three categories genes identified and previously characterized in the literature have been included in the database.

The second block describes molecular adaptation. Genes involved in molecular adaptation to drought stress can be grouped into two categories: (i) genes encoding functional proteins, including protection factors, enzymes for e.g. osmolyte biosynthesis, proteases and genes involved in phospholipid metabolism, detoxification signaling, channels and transporters and (ii) genes encoding regulatory proteins, e.g. involved in gene expression, post-translational modification, signal transduction, hormone signaling and acid anhydride hydrolases.

Layer 2 provides a more detailed categorization of the pathways involved in molecular adaptation of plants to drought stress. For instance, the pathways related to gene expression can be further grouped into four categories: (i) transcription factors, (ii) microRNAs, (iii) histone modification and (iv) chromatin.

Layer 3 finally represents specific gene families involved in drought stress responses. As an example, 10 different drought stress-related transcription factor gene families are represented at this level, among them *DREB*, *bZIP*, *MYC*, *MYB*, *NAC*, *AP2*-domain, *NF-Y*, *ERF*, *WRKY* and zinc fingers.

**Figure 1.**
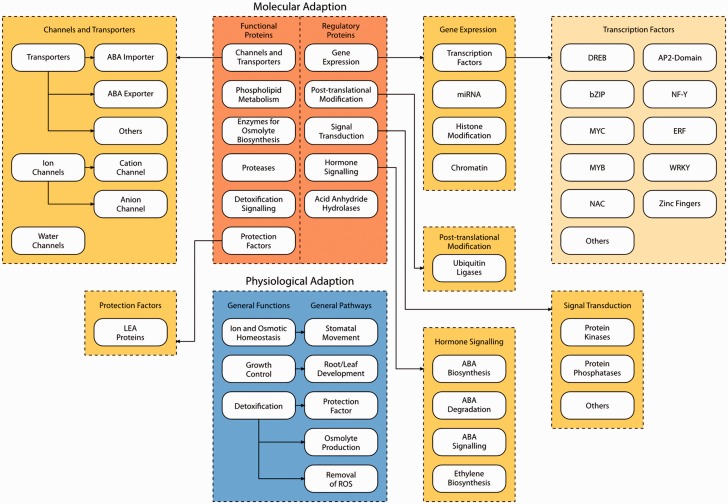
Schematic overview of the three layers of DroughtDB. Layer 1 consists of the two blocks molecular adaptation (orange) and physiological adaptation (blue). Layer 2 (dark yellow) shows a more detailed categorization of the pathways of molecular adaptation (e.g. Gene Expression is grouped into transcription factors, miRNA, Histone Modification and Chromatin). Layer 3 (light yellow) represents specific gene families involved in drought stress response (e.g. transcription factors encoded by the *DREB*, *bZIP*, *MYC*, *MYB*, *NAC*, *AP2*-domain, *NF-Y*, *ERF*, *WRKY* and zinc finger gene family).

To facilitate future dataset upgrades and changes in the visualization of DroughtDB, the data storage and data representation was separated into two distinct components: a database backend and a web frontend. All components and layers (as described before) were modeled into a hierarchical database scheme and implemented in a relational database system. All genes included in the database as well as all orthologs found were interlinked with the corresponding reference genes stored in respective MIPS/PGSB PlantsDB database instances (24). Interlinking allows to retrieve additional information on the respective gene ranging from genome context to sequence information to functional description and Gene Ontology terms and Protein Families domains. In addition, sequence and literature information for the initially used drought response collection extracted from literature can be obtained from DroughtDB. The overall scheme of the three layers is shown in Figure 1.

### Frontend design

The DroughtDB web frontend was implemented in HTML5, CSS3 and JavaScript. It was designed for web browsers based on WebKit and derived rendering-engines (e.g. Firefox, Chrome, Safari). We optimized the DroughtDB site for responsiveness on client systems. All graphics rendering and all interactive features of the website are computed on the client side. To facilitate this we make use of the open-source JavaScript ‘Data Driven Documents’ (D3.js) library. The current implementation of the DroughtDB computes two data objects from the required data stored in the database backend: a JavaScript Object Notation object containing the pathways, and a comma-separated values formatted data object containing genes and related information. These two data objects are transmitted to the client when the website is initially accessed. All further actions in a client’s browser are done based on these datasets. This approach vastly reduces the web- and database-server load by minimizing the number and size of the network requests.

**Figure 2.**
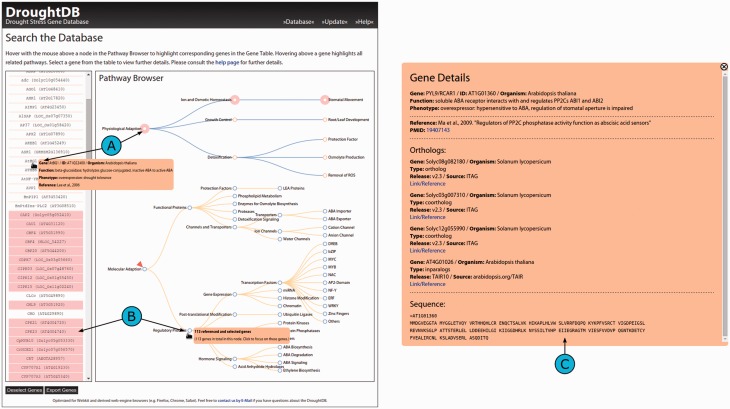
Screenshot of the DroughtDB web interface. (**A**) When hovering above a node in the Pathway Browser corresponding genes are highlighted in the Gene Table. (**B**) Hovering above a gene highlights all related nodes. (**C**) When selecting a gene from the table further details are shown.

The DroughtDB frontend is divided into two adjacent views: the Gene Table and the Pathway Browser (Figure 2A). The Gene Table provides a list of the drought-related genes and initially shows a list of all available gene identifiers. The Pathway Browser is representing the pathways for molecular and physiological adaptation as derived from the publications (Figure 1). The pathways are shown as two tree structures to simplify navigation.

Both views are linked: hovering with the mouse cursor over single gene entries from the Gene Table highlights the corresponding pathways in the Pathway Browser (Figure 2B). Conversely, hovering over a pathway node highlights corresponding genes in the table. Pop-ups in the two views provide additional information. In the Gene Table details about the currently selected gene is shown: its ID, the organism the DB entry was retrieved from, its function, phenotype and the respective reference. In the Pathway Browser the pop-up displays the total number of genes related to a node and, optionally, the number of genes in the current subset.

By clicking onto a node, the list of genes in the Gene Table is reduced to only show the corresponding ones and the node is marked for better recognition (Figure 2B). All navigating features remain unaffected by such a selection; the Gene Table can be reset (i.e. all genes unselected). When clicking onto a gene from the table, a larger static pop-up appears and remains on the screen until it is closed (Figure 2C). Besides the previously described details about a selected gene, it provides additional information that is available in the DroughtDB: links to external references (e.g. the publication the gene was described in) and, if available, orthologs in other organisms. For the orthologs that are also referenced in the MIPS/PGSB PlantsDB links are provided (24).

In addition to a user-oriented help, we offer a user submission system to update and enrich DroughtDB with newly published papers and findings by the research community. A form is provided that allows contributors to enter and submit entries via e-mail.

### Application and data usage

To illustrate functionality and use of DroughtDB two different use cases are described.

Use case 1: the role of *ERA1* (*ENHANCED RESPONSE TO ABA1*, At5g40280) encoding the β-subunit of farnesyltransferase has been well studied in *Arabidopsis.* Deletion of the guard cell-expressed *ERA1* gene leads to an ABA-hypersensitive activation of guard cell anion-channels resulting in stomatal closure (25–27). Double-mutant analyses of *era1* and the ABA-insensitive mutants *abi1* and *abi2* showed a suppression of the ABA-insensitive phenotypes. In line with that, *era1* plants exhibit a reduced transpirational water loss during drought treatment compared with the wild type (26). This control mechanism appears to be very ancient and fundamental. *Arabidopsis* has only a single *ERA1* gene. In all other eight plant model species, the *ERA1* gene is present as a single copy gene, as computed by OrthoMCL (one gene in each species as the orthologous gene, no paralogous genes and no co-orthologous genes). The importance of the *ERA1* gene in engineering drought-tolerant plant has been shown by experiments on manipulating the expression level of the *ERA1* gene in transgenic *Brassica napus* plants (28) and *Triticum aestivum* (wheat) (29). Thus, DroughtDB facilitates both the identification of the respective orthologous gene as well as the corresponding genomic information about *ERA1* orthologous genes by utilizing the pre-computed orthologous relationships between model and crop plants.

Use case 2: *CrNCED1* is a gene encoding the rate-limiting enzyme of ABA synthesis, 9-cis-epoxycarotenoid dioxygenase (*NCED*), isolated from *Citrus reshni* (30). The mRNA levels of *CrNCED1* are upregulated by dehydration and ABA. Transgenic tobacco (*N.* *nudicaulis*) plants constitutively overexpressing *CrNCED1* contain higher ABA levels and display enhanced tolerance to dehydration and drought stress when compared with the wild type. *CrNCED1* is highly similar to more than 22 genes from nine plant species. Interestingly, maize has six orthologous *NCED1* genes, far more than rye (one gene), sorghum (two genes) and rice (three genes), suggesting that maize experienced a major expansion of this ABA synthesis related gene family with possibly newly specified functions and/or expression patterns.

### Discussion and outlook

We implemented a database, DroughtDB, for the representation and comparative analysis of drought-related genes in higher plants. DroughtDB is manually curated and dedicated to molecularly characterized plant genes extracted from literature. Construction of orthologous gene families using nine different model and crop plants including barley and maize facilitates knowledge transfer and helps to identify exclusive pathways and gene functions in plant drought stress response. Interactive access to the database content is provided via a series of intuitive user interfaces as well as batch/bulk download opportunities.

Updates and submissions of new data to DroughtDB are invited and highly encouraged from the expert user communities to further enrich the database content in the future. To assist this critical mission, an intuitive user submission interface was set up, allowing both batch upload and submissions or updates of single gene entities or publications.

As DroughtDB is embedded in the larger framework of MIPS/PGSB PlantsDB (24), the database will directly benefit from the growing amount of plant genome data stored in the system, and will thus also allow to link the information to upcoming important but complex crop plant genomes such as e.g. barley and wheat (18, 31). Orthologous relationships between drought genes and the major gene complements represented within PlantsDB will be calculated on a regular basis allowing knowledge transfer between model and crop plants and may provide new insights into drought-related gene family dynamics. In addition, the collated information of drought stress associated genes in model plants and forthcoming new assays and data in crop plants will allow to also take a comparative perspective and to transcend beyond lists of responsive genes toward a systemic integration and comparative analysis of systemic conservation and diversification.
